# Rhinovirus induces airway remodeling: what are the physiological consequences?

**DOI:** 10.1186/s12931-023-02529-9

**Published:** 2023-09-29

**Authors:** Cassandra Spector, Camden M. De Sanctis, Reynold A. Panettieri, Cynthia J. Koziol-White

**Affiliations:** Rutgers Institute for Translation Medicine and Science, New Brunswick, NJ USA

**Keywords:** Airway remodeling, Rhinovirus, Asthma, Airway epithelium, Airway smooth muscle, Fibroblasts, Myofibroblasts, Airway hyperresponsiveness

## Abstract

**Background:**

Rhinovirus infections commonly evoke asthma exacerbations in children and adults. Recurrent asthma exacerbations are associated with injury-repair responses in the airways that collectively contribute to airway remodeling. The physiological consequences of airway remodeling can manifest as irreversible airway obstruction and diminished responsiveness to bronchodilators. Structural cells of the airway, including epithelial cells, smooth muscle, fibroblasts, myofibroblasts, and adjacent lung vascular endothelial cells represent an understudied and emerging source of cellular and extracellular soluble mediators and matrix components that contribute to airway remodeling in a rhinovirus-evoked inflammatory environment.

**Main body:**

While mechanistic pathways associated with rhinovirus-induced airway remodeling are still not fully characterized, infected airway epithelial cells robustly produce type 2 cytokines and chemokines, as well as pro-angiogenic and fibroblast activating factors that act in a paracrine manner on neighboring airway cells to stimulate remodeling responses. Morphological transformation of structural cells in response to rhinovirus promotes remodeling phenotypes including induction of mucus hypersecretion, epithelial-to-mesenchymal transition, and fibroblast-to-myofibroblast transdifferentiation. Rhinovirus exposure elicits airway hyperresponsiveness contributing to irreversible airway obstruction. This obstruction can occur as a consequence of sub-epithelial thickening mediated by smooth muscle migration and myofibroblast activity, or through independent mechanisms mediated by modulation of the β_2_ agonist receptor activation and its responsiveness to bronchodilators. Differential cellular responses emerge in response to rhinovirus infection that predispose asthmatic individuals to persistent signatures of airway remodeling, including exaggerated type 2 inflammation, enhanced extracellular matrix deposition, and robust production of pro-angiogenic mediators.

**Conclusions:**

Few therapies address symptoms of rhinovirus-induced airway remodeling, though understanding the contribution of structural cells to these processes may elucidate future translational targets to alleviate symptoms of rhinovirus-induced exacerbations.

## Introduction

Rhinoviruses are non-enveloped, single-stranded positive RNA (ss + RNA) viruses that belong to the family *Picornaviridae*, genus *Enterovirus*. There are three major serotypes of rhinovirus (RV), and each utilizes a specific cellular receptor for viral entry – RV-A utilizes intercellular adhesion molecule 1 (ICAM-1), RV-B leverages the low-density lipoprotein receptor (LDLR) for entry, and RV-C binds to and utilizes cadherin related family member 3 (CDHR3). While RVs classically infect human nasal and airway epithelium, RV tropism is not limited to the upper respiratory tract and may infect lower respiratory airways [1]. This may be dependent on RV serotype, as RV-C infection may be more common than RV-A or RV-B in lower respiratory infections [2].

RV infection has been associated with exacerbation of asthma across age groups and RV serotype may determine the severity of symptoms and asthma exacerbations. A strong link between exacerbation of asthma symptoms and RV infection has been observed in multiple patient cohorts [3–6]. Moreover, RV represents the most common cause of exacerbations in children and adults with asthma [7, 8]. Childhood wheeze is associated with RV infection [8] and RV infection may predispose children to developing asthma [9]. However, the severity of RV-induced inflammatory responses in patients with mild to moderate asthma were found to be independent of allergen exposure [10], suggesting virus-specific determinants of exacerbations. In separate cohorts, RV-A and RV-C were more common at all ages and were associated with more severe disease when compared to RV-B [5, 6, 11]. RV-A and RV-C induce stronger immune responses than RV-B, likely due to relatively higher virulence and better ability to replicate in airway epithelial cells (AECs) [5, 12]. Most investigators have utilized RV-A and RV-B strains in experimental investigations, though the identification and emergence of RV-C as an etiological agent of asthma exacerbations in the late 2000’s necessitates its inclusion in future studies.

Because of the large number of prevalent strains across three serotypes, repeated RV infections may occur even within the same year [13, 14]. RV reinfection has been associated with exacerbations of chronic airway disease, including asthma, and was prolonged in patients with chronic airway disease when compared to non-diseased patients [15, 16]. Moreover, a history of asthma and/or atopy may predispose individuals to recurrent RV infection [17]. Respiratory viruses can disrupt the structure and function of airway cells and stimulate wound healing pathways that are protective in healthy tissues, but detrimental in tissues with pre-existing injury or inflammation [18, 19]. Repeated infection in turn amplifies these processes, creating a feed-forward injury-repair loop that induces airway remodeling and the development of irreversible airway obstruction over time. Persistent RV colonization of the respiratory tract after resolution of symptoms as well as asymptomatic infection have also been documented [15, 16, 20, 21] and may contribute to prolonged inflammation or injury repair responses initiated by productive RV infection.

Airway remodeling is a complex process characterized by pathophysiological changes in the morphology and function of airway structural cells and extracellular matrix (ECM) composition. While the term “remodeling” is broad in application, hallmarks of airway remodeling in severe asthma include sub-epithelial fibrosis driven in part by excessive ECM deposition and dysregulation of ECM regulatory enzymes, increased airway smooth muscle mass, disruption of the epithelium including increased proportions of secretory cells and loss of barrier integrity, excessive mucus production, and robust angiogenesis [22–24]. Airway remodeling can be associated with worse clinical outcomes in patients with asthma, and few therapies specifically target its symptoms [25, 26].

T helper cell type 2 (Th2) inflammation present in a majority of asthma cases is thought to be a major driver of airway remodeling. Th2-associated cytokine and chemokine levels have been positively correlated with features of airway remodeling in multiple independent clinical and murine studies [27–31]. RV infection of humans or mice upregulates Th2 inflammatory markers, including interleukin-13 (IL-13), interleukin-25 (IL-25), and interleukin-33 (IL-33) [32–34], which may induce RV-driven exacerbation of asthma symptoms and drive remodeling. Non-Th2 mechanisms may also be responsible for remodeling of asthma airways upon viral infection. While not yet definitively correlated with airway remodeling, recent advances described in this review support a multifactorial etiology for RV-mediated remodeling, including through non-Th2-mediated processes.

While RV mainly infects epithelial cells, growing evidence supports the potential for inadvertent consequences of infection on other structural cells of the airway, including smooth muscle, fibroblasts, myofibroblasts, and endothelium, that may evoke airway remodeling. Structural cells of the airway communicate with each other – and with lung resident and infiltrating immune cells – through the release of soluble cytokines, chemokines, growth factors, and angiogenic factors, as well as ECM proteins and regulatory enzymes to modulate autocrine and paracrine function in response to viral pathogens [35–38]. While the recruitment of immune cells to the site of infection is in many cases necessary for viral clearance, the response of airway structural cells to virus and subsequent contributions to virus-induced remodeling phenotypes – in both the absence and presence of chronic airway disease – has been understudied. This review will outline cellular changes associated with RV-induced airway remodeling in the context of asthma to highlight the often-overlooked importance of airway structural cells for the development of irreversible airway obstruction.

## Structural cells of the airway

**Fig. 1:**
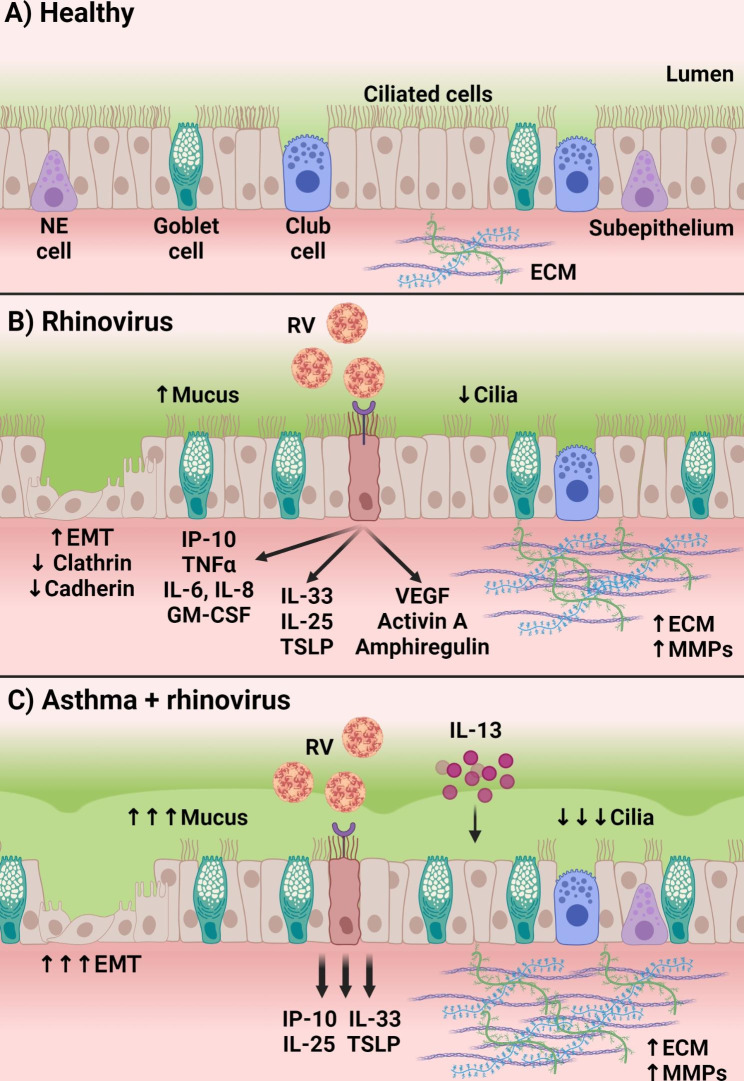
RV infection produces robust remodeling signatures in airway epithelia that are enhanced with asthma. **(A)** The healthy airway epithelial layer is composed of ciliated pseudostratified epithelial cells, goblet cells, neuroendocrine (NE) cells, club cells, and other epithelial cell subtypes that contribute to the overall maintenance of barrier protection and integrity. **(B)** Rhinovirus infection of healthy airways is facilitated by viral receptors on pseudostratified airway epithelia. Infected cells produce proinflammatory type 1 and type 2 cytokines and chemokines, as well as angiogenic soluble factors that are released into the sub-epithelial space. RV infection increases goblet cell number and mucus production and reduces the number of ciliated cells. Epithelial-to-mesenchymal transition (EMT) can be promoted by RV exposure, resulting in morphological changes in epithelial composition. Extracellular matrix (ECM) components and matrix metalloproteinases (MMPs) are upregulated by epithelial cells in response to RV. **(C)** RV infection in the backdrop of existing asthma results in exaggerated responses by airway epithelia, in part due to higher levels of type 2 cytokines, including IL-13. Infected asthma epithelia highly produce type 2 cytokines and chemokines, including IP-10, IL-25, IL-33, and TSLP. Goblet cell number and mucus production are highly enhanced and ciliated cell number is significantly reduced. EMT is induced by RV infection at higher rates in asthma airway epithelial cells. ECM and MMP activity are also highly upregulated in RV infection with asthma. Created with BioRender.com

The airway architecture is comprised of several cell types that coordinate to maintain homeostatic function and structure. Pseudostratified columnar epithelial cells line the airway lumen and provide the first barrier of protection against inhaled particles, environmental toxicants, and pathogens. These epithelia form a selective physical barrier reinforced by tight, gap, and adherens junctions between adjacent cells that, along with secreted mucus, prevent the breach of inhaled insults into the sub-epithelial layers [39]. The airway epithelial layer is composed of a collection of specialized cell types including ciliated columnar cells, goblet cells, basal cells, and others that may become dysregulated by injury or chronic exposure to allergen or inflammation [36, 40, 41]. Importantly, the polarization of airway epithelia facilitates functional secretion of mucus from the apical membrane facing the airway lumen and soluble factors from the basolateral membrane toward the sub-epithelial space (Fig. 1A).

Directly surrounding the airway epithelium is the sub-epithelial space, which contains mesenchymal-like cells including fibroblasts, myofibroblasts, and smooth muscle, as well as ECM that provides structural support to tissues and influences cellular behavior. Airway smooth muscle (ASM) shortening serves as the main driver of luminal narrowing during bronchoconstriction in asthma exacerbations. Healthy ASM is responsive to contractile agonists but in asthma may become excessively sensitive to those stimuli, a phenomenon termed airway hyperresponsiveness (AHR) [42]. The mechanisms controlling viral-induced AHR are not completely understood. Airway fibroblasts and myofibroblasts also reside in the sub-epithelium and are classically thought to produce much of the ECM surrounding the airway - consisting of layers of fibrous collagens, fibronectin, glycosaminoglycans, and proteoglycans [43]. Dysregulation of myofibroblast function in particular has been implicated in mediating airway remodeling through aberrant tissue wound repair mechanisms that contribute to excessive deposition of ECM and contribute to AHR [44]. Finally, blood vessels are situated adjacent to large and small airways, and endothelial cells that line these vessels provide a tight barrier that facilitates blood flow to and from alveoli. Pericytes, vascular fibroblasts, and vascular smooth muscle are also components of lung vessels and have various functions that ensure homeostasis of lung vasculature [45, 46]. Due to the close proximity of small vessels to airways, the lung endothelium may be influenced by pathogen exposure or by angiogenic soluble mediators produced by airway structural cells.

### Epithelial cells

Epithelial cells line the airway lumen and serve as the primary cellular barrier to inhaled allergens, environmental particles, and pathogens. As the first cell layer to come in contact with inhaled RV particles, epithelia are especially important in triggering the initial antiviral response to RV infection. RV exposure of healthy, differentiated AECs triggers pattern recognition receptor activation of antiviral cascades and ultimately results in the transcription of proinflammatory cytokines and chemokines, including type I and III interferons, tumor necrosis factor α (TNFα), interleukin 6, interleukin 8, granulocyte macrophage-colony stimulating factor, and interferon γ-induced protein 10 kDa (IP-10) [47–49]. IP-10 in particular can serve as a reliable proxy for infection given its robust upregulation upon exposure and correlation with viral load [50–53]. In addition to classical antiviral mediators, RV infection of AEC upregulates alarmins IL-25, IL-33, and thymic stromal lymphopoietin (TSLP) that can participate in the recruitment of Th2 cells and type 2 innate lymphoid cells (ILC2s) [48] (Fig. 1B). Additional studies show that RV infection may exacerbate preexisting Th2 inflammation in asthma patients, particularly through the upregulation of Th2 cytokines including IL-33 [33]. RV-A16 stimulation of AECs upregulated production and release of IL-33, which was able to induce T cell and ILC2 activation, as well as Th2 cytokine release [33]. Enhanced IL-33 upregulation in AEC by RV exposure may be due in part to oxidative stress in the airway environment [54]. The duality between the upregulation of antiviral and Th2 mediators by AEC upon RV exposure highlights the balance between appropriate innate responses and pathological injury responses that exist in asthma. Downstream effects of RV-induced Th2 upregulation by AEC are reflected by the effects on other structural cells of the airway, as detailed in later sections, and by the recruitment of a significant number of Th2 immune cells seen in the airways of Th2-high asthma subjects [55].

Upon RV infection, non-diseased AEC has been observed to have gene expression, protein, and morphological changes associated with airway remodeling that, in some studies, was enhanced when compared to AEC from asthma donors. Transcriptional analysis of normal and asthma-derived AECs infected with RV-1A showed upregulated genes encoding ECM components including *FN1* and *ITGB6*, as well as the expected downregulation of genes associated with viral host shut-off mechanisms such as cellular transcription factors, nuclear pore proteins, and RNA-processing genes [56]. However, there was additional differential expression between normal and asthma donors upon RV exposure, with asthma donors showing specific upregulation of genes associated with inflammation and airway repair and remodeling, including *LOXL2* and *MMP10*. In a separate study utilizing fully differentiated AECs infected with RV-A16, there was no difference in antiviral responses between non-asthma and asthma donors, even when the type of asthma was considered (neutrophilic, eosinophilic, pauci-granulocytic, or mixed phenotype), but it was unclear whether remodeling factors were differentially expressed in this model [57].

Airway remodeling in part is mediated by ECM accumulation and dysregulation of ECM regulatory factors. In response to RV-A16 or RV-B2 infection, AECs increased deposition of several ECM components, including perlecan, collagen V, and matrix-bound vascular endothelial growth factor (VEGF) [58]. Paracrine ECM upregulation mediated by airway epithelium may also occur as asthma AECs have been shown to promote upregulation of ECM deposition by fibroblasts when compared to AECs from healthy donors [59]. Tacon et al., demonstrated that ECM remodeling-associated genes including *MMP-10*, *FN1*, and *SERPINE1* were preferentially upregulated in asthma AEC compared to non-asthma-derived AEC [60] (Fig. 1C). RV-A16 or RV-1B induced *MMP9* expression and production within 24 h after exposure of AEC. and was significantly increased in nasal lavage of individuals with confirmed symptomatic RV infection when compared either to baseline after viral clearance or to AEC from uninfected control patients [60]. AEC culture confirmed this effect and identified potential signaling pathways stimulated by RV to promote matrix metalloproteinase 9 (MMP-9) production and overall MMP activity [60, 61] (Fig. 1B). Importantly, formoterol and dexamethasone blocked MMP-9 production induced by RV [62], suggesting that current therapies may reduce RV-induced MMP upregulation expression or activity.

In addition to ECM modulation, RV has been shown to induce epithelial production of angiogenic factors that may act as remodeling mediators on other structural cells of the airway. RV-A16 stimulation of AECs upregulated VEGF, amphiregulin, and activin A [63] that may promote features of airway remodeling [64–66] (Fig. 1B). The same study observed elevated VEGF in nasal lavage samples from patients with confirmed RV infection that was correlated with peak viral titer. VEGF, along with MMP-9, matrix metalloproteinase-10 (MMP-10), tissue inhibitor of metalloproteinases 2 (TIMP-2), and several growth factors, were upregulated in nasal passages of children with RV infection [67], confirming the native upregulation of multiple remodeling signals in response to RV. Moreover, others found that VEGF, fibronectin, and MMP-9 were elevated in nasal lavage fluid from adults with confirmed RV infection [68].

The downregulation of microtubule and ciliary structural genes may be indicative of epithelial-to-mesenchymal transition (EMT) occurring upon RV exposure. EMT of the airway is largely a wound healing or regenerative response triggered by injury to the epithelial barrier and is characterized by the loss of epithelial markers, including E-cadherin and other cell-cell junction markers, and the gain of mesenchymal markers, such as vimentin, fibronectin, N-cadherin, and α-smooth muscle actin (α-SMA) [69, 70]. The trans-differentiation of AECs undergoing EMT ultimately results in a shift from pseudostratified columnar epithelial morphology toward a mesenchymal morphology and cellular behavior that is associated with loss of epithelial barrier function [69, 71, 72]. Importantly, Th2 inflammation may upregulate mechanistic signaling associated with the promotion of EMT in asthma airways [73–76]. Though EMT is indicative of wound healing processes, chronic insults such as Th2 inflammation or inhaled factors may result in long term dysregulation of the epithelial barrier that contributes to airway remodeling. Because AECs are the primary physical and immunological airway barrier, EMT and accompanying breakdown of the epithelial layer may predispose underlying airway tissue to further damage.

RV exposure can also induce EMT and associated signaling pathways. BEAS-2B cells infected with RV-A16 displayed characteristics of EMT including reduced cadherin and cytokeratin cell junction proteins, increased fibronectin and vimentin, and mesenchymal-like cellular morphology by 120 h post-infection [77]. In primary air liquid interface (ALI)-differentiated AECs, the inflammatory environment prior to RV-A16 exposure determined cellular progression toward EMT versus a mucus metaplastic cellular phenotype (Fig. 1C). Pretreatment with transforming growth factor-β (TGFβ), which modeled a pro-fibrotic environment, upregulated EMT-associated gene expression and ICAM-1 [77]. RV-A16 infection reduced cilia-associated structural gene expression in a manner resembling that of IL-13 pretreatment, suggesting EMT in response to RV may also be associated with the dysfunction of cellular injury responses in epithelia [57]. In an injury-repair model of ALI-differentiated AECs characterized by incomplete polarization of individual cells, RV-A39 infection impeded further repolarization of the epithelial layer [78]. Interrupted repolarization upon RV exposure was identified by aberrant Crumbs cell polarity complex component polarity marker distribution and diminished occludin and E-cadherin at the periphery of infected cells [78]. This report suggests prior injury from chronic airway inflammation or disease may predispose RV-infected epithelia to EMT and overall epithelial barrier dysfunction.

Current evidence suggests a RV infection can skew epithelial layer cell composition toward hypersecretory, goblet cell-biased phenotypes that are enhanced in pre-existing asthma environments. Increased goblet cell frequency and excessive mucus production is characteristic of airway remodeling in chronic airway disease and can contribute to severe airway obstruction [79–83]. Even in the absence of viral infection, goblet cell and mucus metaplasia have been well-documented to occur in asthma epithelia in vitro and in clinical studies [79, 84, 85] and has been hypothesized to be a consequence of the Th2 immune environment in asthma. For example, in an in vitro model using ALI-differentiated AECs, IL-13 treatment alone increased goblet cell number and reduced ciliated cell number, in addition to inducing elevated mucus production [86]. Th2 cytokine exposure of AECs stimulated transcription of *SPDEF*, *FOXA3*, and *RUNX2* genes that drive and regulate goblet cell differentiation of epithelia and ultimately promote robust *MUC5AC* expression, goblet cell hyperplasia, and mucin production [87, 88].

Using RNA-seq approaches, investigators reported differential expression of genes associated with epithelial structure and morphology between non-asthma and asthma in RV-A16-infected ALI-differentiated AECs [89]. Microtubule and ciliary genes including *DNAH6*, *DNAI1*, and others were downregulated, while *MUC5AC* was upregulated preferentially in asthma-derived AEC [89]. The downregulation of cilia structural genes and upregulation of secretory-associated genes suggests an induced bias toward hypersecretory epithelial phenotype. Additional studies have found that a pre-existing Th2 immune environment alone consisting of IL-13 or interleukin-4 (IL-4) exposure was sufficient to modulate expression of remodeling genes including *MUC5AC* and *FGF2* [57, 90].

Similar functional and molecular processes were demonstrated upon RV infection of AECs. RV-A16 infection of ALI-differentiated AEC resulted in elevated mucus production indicative of mucus metaplasia and was accompanied by increased goblet cell frequency and reduced ciliated cell number [86, 90] (Fig. 1B). Several reports have corroborated *MUC5AC* upregulation and mucin hypersecretion by epithelia upon exposure with strains representing all three RV serotypes [91–95]. In primary AEC, *FOXA3* was upregulated by RV-A16 or RV-1B exposure at comparable levels as induced by IL-13 [96]. RV-induced *FOXA3* upregulation was accompanied by *FOXA3*-dependent abrogation of antiviral responses in exposed cells, including and the inhibition of interferon and interferon-stimulated gene signaling. In mice, RV infection resulted in goblet cell metaplasia, poor interferon β production, and attenuated viral clearance that was absent in *FOXA3*^*−/−*^ animals [96].

Goblet cell hyperplasia is a hallmark of Th2 inflammation that is largely driven by IL-13 [97–99]. The shift toward secretory phenotype may represent a disruption of the antiviral response produced by airway epithelia upon RV exposure toward a Th2-biased inflammatory environment that prevents viral clearance and enables prolonged RV-mediated goblet cell hyperplasia. In Th2-primed environments, such as asthma airways, RV exposure may promote exuberant hyperplasia contributing to worsened airway occlusion (Fig. 1C).

### Smooth muscle

In airway remodeling in asthma, smooth muscle is a main effector cell driving airway obstruction. Remodeling associated with severe asthma has been characterized by sub-epithelial thickening that is, in part, due to greater smooth muscle mass surrounding the airway [100]. In chronic asthma, increased ASM contraction and proliferation drive the mechanical compression of the airway and mediate luminal narrowing. As direct mediators of AHR, enhanced ASM contraction, hypertrophy, and hyperplasia thus propagate pathological airway remodeling and lung function decline, even in the absence of inflammatory cell infiltration [101–103]. Despite observations asserting the strong association between smooth muscle hypertrophy and/or hyperplasia in asthma, it is not fully understood how RV may mechanistically augment ASM function. The understood role of smooth muscle in remodeling suggests a two-fold mechanistic contribution to airway obstruction: sub-epithelial thickening due to induced ASM migration, ASM proliferation, and ECM deposition within the sub-epithelial layer; or, induced AHR independent of sub-epithelial thickening.

**Fig. 2:**
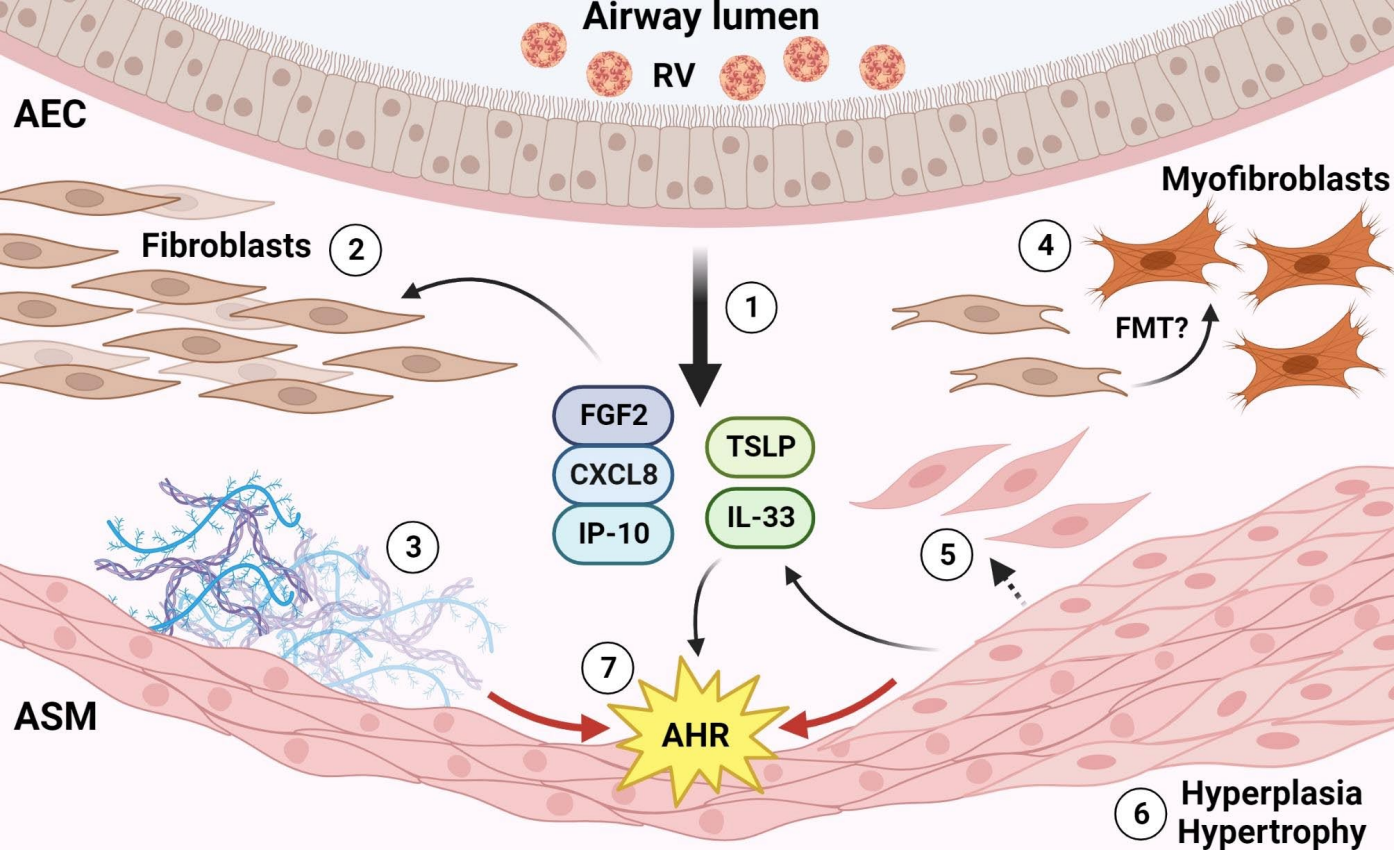
Effects of RV infection on airway smooth muscle and fibroblasts. Exposure of RV on airway epithelium results in downstream responses that augment migration, proliferation, and cellular function of sub-epithelial structural cell types. **(1)** Infected airway epithelial cells (AEC) produce FGF2, CXCL8, IP-10, and alarmins such as IL-33 and TSLP that act on airway fibroblasts and airway smooth muscle (ASM) to induce sub-epithelial thickening, cell proliferation, and mobilization surrounding the airway. Smooth muscle may also produce IL-33 that contributes to remodeling-associated cytokines induced by RV infection. **(2)** Fibroblasts accumulation with RV infection occurs in response to FGF2 and CXCL8 along with **(3)** smooth muscle migration through the sub-epithelial layer. **(4)** RV infection may promote fibroblast-to-myofibroblast transition (FMT), resulting in increased myofibroblast numbers surrounding the airway. **(5)** Extracellular matrix deposition by smooth muscle and fibroblasts, as well as MMP activity, is upregulated during RV infection. **(6)** RV infection of the airway induces smooth muscle hyperplasia and hypertrophy. **(7)** Altogether, airway RV exposure promotes airway hyperresponsiveness (AHR) in a multifactorial manner mediated by both sub-epithelial thickening and augmentation of contraction and relaxation responses in airway smooth muscle. Created with BioRender.com

Chemokines produced upon RV infection of airway epithelium may promote ASM migration toward the epithelial layer and potentially contribute to sub-epithelial thickening of the airway. Infection of ALI-differentiated AEC infected with RV-A16 elicited robust production of IP-10, C-C motif chemokine ligand 5 (CCL5), and CXC motif chemokine ligand 8 (CXCL8) [104, 105] (Fig. 2). Shariff et al., demonstrated migration of ASM in response to AEC RV-A16-conditioned medium that could be recapitulated by exogenous treatment of CCL5, CXCR8, or IP-10 [104]. Of note, AEC production of chemokines in response to RV was dependent on viral replication, suggesting that increased susceptibility to infection may promote enhanced migratory effects on ASM. Similarly, Celle et al., showed that RV-A16-infected AEC medium transfer to ASM induced IP-10/CXC motif chemokine receptor 3 (CXCR3)-dependent ASM migration through microchambers that was significantly enhanced in asthma ASM versus non-asthma ASM due to the increased distribution of the growth-inhibitory CXCR3-B isoform in severe asthma subjects [105]. While burgeoning reports of ASM migration support that RV infection may induce ASM-mediated sub-epithelial thickening, there have been few reports on mechanistic aspects of ASM proliferation or hypertrophy are specifically induced by RV exposure. One such study utilized a murine RV-1B infection model and observed robust ASM thickening upon repeated infection that was further exacerbated when combined with allergen exposure [106].

While many pathways activated by epithelial soluble mediators proceed through inflammatory pathways, it is unclear if events of asthma exacerbation and asthma severity proceed through, or are independent of, Th2-mediated inflammation. The Th2 cytokine IL-33 has emerged as a potential smooth muscle effector of remodeling and AHR in asthma due to its association with sub-epithelial thickening and ECM deposition in asthma [27, 30]. Murine studies utilizing RV challenge after early life exposure to cockroach extract allergen or pneumonia virus of mice (PVM) yielded elevated IL-33 production upon RV-1B exposure, but there was little effect on ASM mass [107]. However, a related study authored by the same research group showed that repeated allergen and PVM exposure elicited significantly increased ASM mass and collagen deposition [108], suggesting that frequent or chronic exposure to pathogen and allergen may be required for smooth muscle hyperplasia in respiratory viral infection.

While the cellular source of IL-33 exposure of smooth muscle is canonically thought to be from RV-infected airway epithelium or lung immune cells, smooth muscle may also produce IL-33 in response to direct RV exposure. IL-33 gene expression was upregulated in ASM upon exposure to conditioned medium from double stranded RNA (dsRNA)-treated AECs or direct exposure of dsRNA or RV-1B [109] (Fig. 2). Subsequent in vitro studies using primary ASM from mild to moderate asthma subjects demonstrated elevated gene and protein expression of baseline and RV-1B-evoked IL-33 when compared to healthy controls [110]. Regulation of IL-33 largely occurred through toll-like receptor 3 (TLR3), and downstream through transforming growth factor-β-activated kinase, suggesting Th2 enhancement may result in smooth muscle reactivity and that smooth muscle may produce some innate immune responses in the presence of free virus. Additionally, RV-induced smooth muscle production of IL-33 may act in an autocrine or paracrine manner to promote smooth muscle-driven remodeling phenotypes within the airway, though these potential mechanisms have not been characterized.

ASM may contribute to remodeling activity and phenotypes in the airway upon direct RV exposure, though few studies have specifically investigated these mechanisms. Of these studies, there is an emphasis on the differential ECM deposition between non-asthma and asthma derived ASM. Direct exposure of non-asthma and asthma-derived ASM monoculture to RV-A16 or RV-A2 yielded differential induction of ECM components observed in remodeled airways. While RV exposure induced fibronectin and perlecan production in non-asthma-derived ASM, asthma-derived ASM had enhanced deposition of fibronectin and collagen IV, but not perlecan [111] (Fig. 2). Collagen deposition and accumulation by ASM has been linked to β_2_ adrenergic receptor (β_2_AR) agonist and glucocorticoid resistance [112], which may contribute to smooth muscle bronchodilator insensitivity seen in remodeled airways.

RV-induced AHR and attenuation of ASM relaxation independent of sub-epithelial thickening serves as an alternate mechanism of luminal narrowing in airway remodeling. ASM contraction has been established to proceed through either calcium-dependent or calcium-independent (sensitization) pathways [113]. The involvement of RV in AHR has been established [114], though evidence of ASM hyperresponsiveness related to airway remodeling continues to accumulate (Fig. 2). In addition to AHR present in asthma and following RV infection is the occurrence of attenuated relaxation responses to inhaled bronchodilators, such as β_2_AR agonists. A series of in vitro studies utilizing AEC-ASM co-culture or ASM alone have established the role of RV in abrogating β_2_AR function in ASM. First suggested by Hakonarson et al., exposure of rabbit or human ASM to RV-16 attenuated β_2_AR-driven ASM relaxation to isoproterenol [115]. Changes in ASM responsivity can align with clinical evidence of diminished β_2_AR agonist efficacy in the event of asthma exacerbations [116]. Subsequently, ASM exposure to RV-A16 conditioned medium was found to attenuate isoproterenol-induced cAMP generation, confirming dysregulation of β_2_AR signaling by RV [117]. Multiple studies have since observed RV-dependent resistance to bronchodilators spanning diverse mechanistic pathways [118–121]. Novel ex vivo studies utilizing human precision cut lung slices (hPCLS) have demonstrated that RV-C15 exposure augments both carbachol-induced bronchoconstriction and β_2_AR agonist-induced relaxation, as well as the release of inflammatory mediator IP-10 and macrophage inflammatory protein-1β [51, 118]. hPCLS responsivity, defined as agonist-induced tissue constriction and inflammatory response, was independent of RV-C15 viral load [51]. The attenuation of airway relaxation by pharmacological intervention in response to RV exposure may further contribute to luminal narrowing and obstruction promoted by airway remodeling. Whether these changes in airway tone following RV exposure are due to remodeling of the airways, or due to increased release of remodeling factors, is unclear.

### Fibroblasts

A hallmark of airway remodeling is airway wall thickening that is in part due to enhanced activation, proliferation, and migration of fibroblasts within sub-epithelial tissue, as well as differentiation of fibroblasts into contractile myofibroblasts [122]. While the contribution of fibroblasts to airway remodeling in asthma has been established [122], few studies have specifically explored the effects of airway RV infection on lung resident fibroblasts. These studies describe the functional effects of factors produced by infected epithelia or direct infection on fibroblast activity and function in airway remodeling.

Sub-epithelial fibroblast accumulation due to proliferation and migration may be promoted by soluble mediators released by RV-infected epithelium. AEC infected with RV-A16 or RV1B released fibroblast growth factor 2 (FGF2) that was proportional to the number of virions used in exposure [61]. FGF2 is a potent growth factor that regulates migration and proliferation of airway fibroblasts, myofibroblasts, and smooth muscle and is released from injured epithelia [123–125]. BEAS-2B infection with RV-1B, and subsequent conditioned medium transfer to lung fibroblasts, induced fibroblast proliferation that was partly dependent on the availability of FGF2 [61] (Fig. 2). Clinically, FGF2 was elevated in nasal aspirates of patients with confirmed RV infection compared to uninfected baseline [61]. Additionally, fibroblasts may be recruited to, and accumulate in, sub-epithelial airway layers upon RV infection. Primary fibroblast migration within a Boyden chamber was robustly induced upon exposure to RV-A16-infected AEC conditioned medium. The magnitude of this effect was dependent on length of treatment and was dependent on the presence of CXCL8 and IP-10 in AEC conditioned medium. Moreover, the cognate receptors CXC motif chemokine receptor 1 (CXCR1) and CXCR3 were expressed by and detected on fibroblasts, indicating a chemokine-dependent mechanism of fibroblast migration in response to RV infection in the airway [126].

While RV mainly infects airway epithelia, select studies exploring airway remodeling have addressed whether direct infection of primary lung fibroblasts or cell lines modulates fibroblast function. The epithelial barrier may lose integrity upon infection due to tight junction dysregulation leading to paracellular permeability and the potential transmigration of cell-free viral particles [127, 128]. Productive infection of primary lung fibroblasts with RV-A16 in vitro has been reported [129]. However, studies directly infecting fibroblasts may not be widely applicable to all RV strains due to the utilization of serotype-specific viral entry receptors; namely, RV-C strains utilize CDHR3 as an entry receptor, which imparts a narrow tropism as a consequence of its exclusive expression in ciliated airway epithelia [130, 131]. Infection of HLF1 or WI38 fibroblast cell lines with RV-A16 or RV-A2 induced upregulation of *TGFB1*, *MMP9*, *COL1A1*, *ADAM33*, *CHI3L1*, *LTC4S*, and *ACTA2* [132]. These genes have been associated with an airway remodeling phenotype and suggest activation of a robust remodeling response upon RV exposure. Remodeling gene upregulation was possibly due to oxidative stress induced by infection. In primary human lung fibroblasts, RV-A16 increased TGFβ and arginase production that could be blocked by NADPH oxidase inhibition [133]. Normal and asthma-derived lung fibroblasts have been observed to mount rapid proinflammatory and protective responses when exposed to RV-1B [134], which may facilitate the observed activation of remodeling pathways.

Clinical assessment of the airways of asthma subjects with respect to remodeling consistently shows sub-epithelial fibrosis, which is likely mediated by increased ECM deposition upon fibroblast activation [135, 136]. Fibroblasts are responsive to soluble signaling factors produced by asthma AECs, as in vitro transfer of asthma AEC conditioned medium to normal fibroblasts induced robust upregulation of *COL1A1, COL3A1*, and *FN1* that was not observed in non-asthma AEC conditioned medium transfer [59]. RV may promote fibroblast activation or further exaggerate ECM deposition, as observed in asthma subjects. Nasal polyp fibroblast exposure to RV-A16 elicited robust upregulation of matrix metalloproteinase 2 (MMP-2) and MMP-9 regulatory enzymes, indicating disruption of the ECM in response to virus [137]. Primary human lung fibroblasts exposed to RV-A16 or RV-B2 had increased expression of ECM components including perlecan and collagen V. This was confirmed in vivo in lung tissue of C57/BL6 mice infected with RV-1B, where fibronectin and Col1A1 were significantly upregulated within 24 hours post-infection [58]. ECM upregulation in vitro was potentially mediated through TLR pathway activation, as polyinosinic:polycytidylic acid (poly I:C) and imiquimod – TLR-3 and toll-like receptor 7/8 agonists, respectively – induced robust ECM production in fibroblasts [58]. Furthermore, recurrent RV-1B infections in mice (twice per week for three weeks) in the absence of allergen induced sub-epithelial thickening and robust collagen deposition in sub-epithelial layers of airways in vivo [106]. These data have yet to be reproduced in human studies, though the ability of RV to promote fibroblast-targeting release of soluble mediators, the upregulation of remodeling factors by fibroblasts exposed to RV, and the proclivity of airway fibroblasts to activate and deposit ECM in the presence of asthma-associated stimuli [59, 122, 136] suggests a strong likelihood for enhanced ECM deposition upon native RV infection.

### Myofibroblasts

Myofibroblasts may abnormally accumulate in the sub-epithelial layer along with fibroblasts and smooth muscle cells in remodeled asthma airways [138, 139]. Myofibroblasts are contractile cells that exhibit enhanced ECM deposition and also contain specialized adhesion complexes that join cytoplasmic microfilaments to ECM components [140]. These intracellular-ECM interactions provide enhanced tensile strength that may function to repair wounds or, in the case of airway hyperresponsiveness, induce and evoke contractile responses in the presence of allergic or pathogenic stimuli [44, 141]. Additionally, in pathologies with increased ECM deposition, such as asthma, myofibroblast accumulation may further dampen the elastic recoil of lung tissue and potentiate contraction of stimulated airways [142, 143].

Through a process called fibroblast-to-myofibroblast transition (FMT), airway fibroblasts exposed to extracellular stimuli or mechanical stress may become active and proliferate or take on a transitional phenotype that proceeds to mature myofibroblast differentiation [143]. Experimentally, the treatment of human fibroblasts with TGFβ promotes FMT characterized by robust α-SMA upregulation and the formation of stress fibers as well as increased ECM deposition and contractility of the cells [144–146]. While molecular signatures of myofibroblast differentiation may appear upon short exposures to stimuli [147], long-term TGFβ treatment of WI-38 fibroblasts over 20 days induced both molecular and morphological features of FMT as well as the upregulation of remodeling-associated genes, including *FN1*, *TIMP1*, *ITGB1*, and multiple *MMP*s [148].

Myofibroblasts may be more permissive to RV infection and replication than their fibroblast counterparts. Fibroblasts exposed to TGFβ to promote FMT exhibited enhanced RV-A16 infection and replication that was further amplified in cells from asthma donors. The inhibitory effect of TGFβ on antiviral interferon I responses in infected cells could promote RV replication [149]. Still, the question remains if RV infection can induce FMT in exposed airways (Fig. 2). While this proposition has not been explored, Sugiura et al., demonstrated that poly I:C exposure could induce FMT in a dose-dependent manner in fibroblast cell lines and in primary human lung fibroblasts. In this report, poly I:C-induced FMT was dependent on TGFβ produced by the treated cells, which increased production of fibronectin and collagen I [150]. While poly I:C is a potent ligand of TLR-3 and may exaggerate physiological responses, the induction of FMT in in vitro fibroblasts demonstrates the potential for RV to trigger myofibroblast trans-differentiation, and induction of remodeling signatures in the airway upon direct exposure to virus.

### Endothelium and vasculature

Angiogenesis and vascular changes associated with remodeling have been attributed to angiogenic factors present in asthma airway environments. The sub-epithelial layer of asthma airways show increased vascular density [151, 152] and exhibit elevated permeability that may render airways vulnerable to edema or infiltration by circulating immune cells [153, 154]. Together, these events may exacerbate swelling and inflammation of neighboring airways and contribute to obstruction. However, few studies have addressed the effect of RV on angiogenesis. As endothelial cells are not normally exposed to inhaled viral particles, RV infection may affect endothelia indirectly through the exposure of soluble factors produced by infected epithelia or secondary factors from other submucosal cell types. Direct infection may be possible if loss of airway and vascular barrier integrity occurs, and free viral particles then encounter the endothelium.

Endothelial cells are responsive to several angiogenic growth factors and cytokines that are also involved in airway remodeling, including VEGF, angiogenin, platelet-derived growth factors, and FGF2 [155, 156]. In response to cytokine and chemokine stimuli, lung-derived endothelial layers may lose membrane integrity and be permissive to increased immune cell adhesion and trans-endothelial migration [157]. Airway epithelial cells highly produce remodeling factors in response to RV exposure [58, 61, 158] which may act to indirectly stimulate remodeling responses in airway-adjacent endothelia. Additionally, soluble factors produced by smooth muscle, fibroblasts, and myofibroblasts may contribute to the pool of endothelial remodeling effectors. In particular, the robust induction of VEGF production upon RV-A16 exposure of epithelial cells has a large implication on the potential of RV to indirectly induce airway remodeling though endothelial cell stimulation [63, 158]. The potent effects of excess VEGF on microvascular remodeling and angiogenic processes in asthma and airway disease have been reviewed [65, 159].

A paucity of information exists that suggests RV can infect endothelium, though transient productive RV infection has been reported in vitro for the RV-A serotype, which utilizes surface ICAM-1 as an entry receptor [160, 161]. Using in vitro infection models, recent studies have characterized the effects of RV exposure of endothelial cells, including the impairment of migratory and proliferative abilities, promotion of vascular permeability, and robust production of pro-inflammatory and remodeling-associated factors. Lung-derived human microvascular endothelial cells (HMVEC-Ls) demonstrated distinct loss of endothelial barrier integrity resulting in depleted cadherin expression and induced permeability after RV-A16 exposure. This may be associated with increased apoptosis that was also observed or possibly broad dysfunction of the endothelium that transiently prevented proliferative and migratory capacity of exposed cells [162, 163]. Vascular permeability in response to RV exposure may enable trans-endothelial migration of circulating immune cells into airway-adjacent tissue and may exaggerate airway inflammation [164].

Upon RV exposure, HMVEC-Ls also produced strong inflammatory responses, including Th2 cytokines IL-4 and IL-13, that may promote remodeling cascades within lung microvasculature or other airway structural cells [161]. Notably, direct infection of HMVEC-Ls induced the upregulation of *VEGFA*, *ANGPT1* (angiopoietin-1), and *FGF2* gene expression, as well as upregulation of VEGF receptor genes *FLT1*, *KDR*, and *NRP1* [163]. Importantly, the upregulation of both proangiogenic mediators and receptors in HMVEC-Ls may enable a feed-forward signaling loop that promotes endothelial remodeling and neovascularization near airways. In addition to the potential exposure to high concentrations of these soluble remodeling factors produced by infected airway epithelia or other structural cells, the simultaneous upregulation of these genes in endothelial cells may represent an autocrine mechanism of vascular remodeling induced by RV-associated signaling.

## Therapeutic approaches for rhinovirus-induced airway remodeling

The complex activities and interactions among structural cells of the airway as described in this review highlight the challenges of treating or preventing RV-induced airway remodeling. Nevertheless, current asthma therapies may alleviate some factors of remodeling due to the broad reduction of airway inflammation. Commonly prescribed asthma therapies include: short-acting β-agonists and muscarinic antagonists, which are used to relieve acute symptoms; long-acting β-agonists used to control symptoms; and inhaled corticosteroids (ICS) used to control asthma through prevention of exacerbations by dampening lung inflammation. Despite continual evolution of therapies targeting asthma symptoms – including features of airway remodeling – few clinical studies have investigated endpoints directly related to RV-induced airway remodeling and represents an important unmet need in future therapeutics development. While remodeling-associated endpoints have been observed in clinical studies – such as elevated VEGF in nasal lavage of both non-asthma adults and children with asthma upon natural RV infection – these studies lack the additional factor of ICS or other therapy use [63, 158]. One study that observed natural RV infection in adults with asthma documented prior use of ICS for enrolled patients, though the overall endpoints of the study did not focus on features of airway remodeling in response to RV infection [3].

Still, experimental evidence of the utility of existing therapies to downregulate remodeling-associated responses supports their potential use to mitigate RV-induced airway remodeling. ICS such as dexamethasone, fluticasone, and budesonide are known to downregulate Th2 inflammation in asthma, and consequently, features of remodeling driven by structural cells of the airway including AEC, ASM, and fibroblasts. While no clinical studies have yet sought to pharmacologically inhibit the effects of RV-induced goblet cell and mucus upregulation, murine in vivo studies using glucocorticoids have demonstrated the ability to abrogate allergen-induced goblet cell hyperplasia and downregulate allergen-induced *MUC5AC* expression [165, 166]. The success of glucocorticoids in reversing goblet cell hyperplasia in these models was largely attributed to the blockade of IL-13 or nuclear factor-κB signaling known to potentiate goblet cell differentiation and mucin synthesis. Recently, in vitro studies using ALI-differentiated AECs observed the ability of the muscarinic antagonist tiotropium or the ICS fluticasone propionate to decrease RV-A16- or RV-1B-induced mucin production and goblet cell hyperplasia [167]. Combined treatment of ICS with a β_2_AR agonist reduced RV-induced epithelial production of the chemokines CCL5, CXCL8, and IP-10, VEGF, and FGF2 in vitro [168, 169], highlighting the success of combination therapy in managing asthma exacerbations and airway remodeling lesions.

Likewise, ICS can prevent sub-epithelial thickening mediated by smooth muscle in asthma airway remodeling [170], though no studies have yet shown the ability of ICS to prevent RV-induced AHR, reverse attenuation of relaxation, reduce ECM deposition, or reduce proinflammatory responses elicited by ASM. ICS may prevent fibroblast proliferation in asthma environments [171], though even less is known about how ICS may influence the effects of RV in these cell types in native tissues. Though ICS have been successful in reducing asthma symptoms and remodeling lesions, RV-induced asthma exacerbations may be resistant to ICS [172, 173], as between 5 and 10% of asthma patients are unresponsive to ICS therapy [174]. Non-ICS therapies for asthma may also reduce features of airway remodeling, though it is unclear if they are effective in preventing additional effects from RV infection.

Antiviral therapies that directly inhibit phases of the RV replication cycle have been investigated as treatment options, though few have been successful in clinical trials. Of the few antivirals to be tested in humans, viral entry inhibitors pirodavir, pleconaril, tremacamra, and vapendavir generally reduced the duration of symptoms but either did not provide an overall benefit or did not meet desired study endpoints [175–179]. Of these antivirals, only vependavir was tested in patients with asthma [175]. Pleconaril was the most successful antiviral in clinical trials to date but has not been approved for use in part because it increased cytochrome P450 enzyme activity that predisposed to risk for adverse drug interactions [177]. Moreover, none of the existing RV antivirals have been tested to inhibit symptoms of airway remodeling.

Evidence suggests that biologics may reduce RV infection severity and duration in allergic asthma. Many biologics tested in the context of severe asthma target immune cells or Th2 cytokine signaling with the aim of reducing inflammation and potentially remodeling [180]. Notably, the anti-IgE monoclonal antibody omalizumab reduced symptom duration and viral shedding in a cohort of children with RV-induced exacerbations [181]. Long term use of omalizumab for severe asthma was able to reduce features of airway remodeling associated with exacerbations, including decreases in MMP production, airway inflammation, and basement membrane thickness [182–184]. As IL-33 appears to play a role in inflammatory responses of the lung following RV exposure, newer biologic therapies targeting IL-33 signaling, such as astegolimab and itepekimab, may also be effective in preventing RV-induced asthma exacerbations and subsequent airway remodeling [180, 185, 186]. Table 1 summarizes evidence of potential therapies that may prevent or ameliorate RV-induced airway remodeling discussed in this section.

**Table 1:** Potential therapies for rhinovirus-induced airway remodeling

Drug class	Therapeutic	Mechanism of action	Effect on airway remodeling signatures
Inhaled corticosteroid (ICS)	Dexamethasone	Glucocorticoid receptor agonist	Blockade of RV-induced MMP-9 production by ALI-differentiated AECs in vitro [62]; no clinical trial data to date
	Fluticasone proprionate	Glucocorticoid receptor agonist	Reduction of RV-induced goblet cell hyperplasia and mucin production in vitro [167]; reduction of RV-induced VEGF in BEAS-2B cells when used alone and reduction of FGF2 when in combination with salmeterol [169]; no clinical trial data to date
	Budesonide	Glucocorticoid receptor agonist	Suppression of RV-induced CXCL8, IP-10, and VEGF production by AECs in vitro [168]; reduction of MMP-8 production in mild-to-moderate asthmatic children [191]; attenuation of allergen-induced myofibroblast proliferation and reduction of AHR in mild asthmatic adults when used in combination with formoterol [192]
Anti-muscarinic	Tiotropium bromide	Long-acting muscarinic receptor antagonist	Modest reduction of RV-induced goblet cell hyperplasia and mucin production in vitro [167]; no clinical trial data to date
Long-acting beta agonist	Formoterol	β_2_ adrenergic receptor agonist	Reduction of *MMP9* expression in BEAS-2B cells [62]; no clinical trial data of formoterol monotherapy to date
Antiviral	Pirodavir	Inhibits viral capsid uptake and RNA uncoating	Modest reduction of RV symptoms in healthy subjects [176]; effect on RV-induced airway remodeling not studied
	Pleconaril	Inhibits viral capsid cellular attachment and RNA uncoating	Reduction in length and severity of RV symptoms in healthy subjects [177]; effect on RV-induced airway remodeling not studied
	Tremacamra	Viral entry inhibitor	Reduction of RV symptom severity in healthy subjects [178]; effect on RV-induced airway remodeling not studied
	Vapendavir	Inhibits viral capsid uptake and RNA uncoating	Reduction in length and severity of RV symptoms in asthmatic subjects [179]; effect on RV-induced airway remodeling not studied
Biologic	Omalizumab	Anti-IgE monoclonal antibody	Reduction in length and severity of RV symptoms, reduced viral shedding, reduced frequency of RV infection, and reduced occurrence of acute exacerbation in children with allergic asthma [181, 193]; decreased MMP concentration in bronchoalveolar lavage fluid, reduced acute exacerbation frequency, decrease in reticular basement membrane thickness in adult subjects with severe asthma [182–184]
	Astegolimab	Anti-IL-33 monoclonal antibody	Reduced acute exacerbations in adults with severe asthma [185]; effect on airway remodeling not studied
	Itepekimab	Anti-IL-33 monoclonal antibody	Reduced acute exacerbations and improved asthma-related quality of life in adults with moderate-to-severe asthma [186]; effect on airway remodeling not studied

## Conclusions

For some, RV is a seasonal common cold that presents as transient illness. For individuals with asthma, RV infection my become persistent, reoccurring, and exacerbate asthma symptoms such that they become uncontrolled by maintenance therapies. Asthma predisposes individuals to airway remodeling that leads to irreversible airway obstruction through the hypersecretion of mucus and the thickening of the sub-epithelial layer mediated by airway smooth muscle hyperplasia and hypertrophy as well as fibroblast and myofibroblast proliferation. This review has focused on the physiologic responses of airway structural cells to RV exposure and associated RV-evoked remodeling phenotypes.

Rhinovirus infection of the airway epithelia results in intraepithelial immune activation and cellular stress. Infected epithelia produce soluble mediators that may act on neighboring sub-epithelial cells to promote pathophysiological features of airway remodeling. Non-epithelial structural cells of the airway have been understudied regarding their respective roles in remodeling, as well as their contributions to the lasting impact on airway function following RV infection. Moreover, differential cellular responses to RV between asthma and non-asthma subjects, and in multiple patient-derived cell types, support that RV infection can amplify and exaggerate existing asthma-associated inflammation and aberrantly activate wound healing pathways.

Despite the seasonal prevalence of RV and its frequent recurrence, factors promoting predisposition to RV-induced airway remodeling are not fully understood. However, genetic and epigenetic factors may determine in part which individuals may experience enhanced airway remodeling in response to viral infection. A recent review has compiled data describing that individuals with asthma may be predisposed to features of airway remodeling based on single nucleotide polymorphisms in remodeling-associated genes, including *IL13*, *PLAUR*, *VEGFA*, and *CHI3L1* [187]. Additionally, a minor allelic variant within the RV-C15 receptor CDHR3 may predispose individuals to RV susceptibility. The rs6967330 A allele in CDHR3 that has been previously demonstrated to be an asthma-risk allele has also been associated with higher RV viral load, RV protein expression, and increased ciliogenesis in AECs [188]. This CDHR3 allele has not yet been associated with canonical features of airway remodeling but viral susceptibility and increased asthma risk may play a role in the predisposition to remodeling features. Furthermore, there is evidence indicating that RV infection increased methylation of genomic DNA in nasal epithelia from asthma donors but not non-asthma donors and was reported alongside data showing differential DNA methylation patterns between non-asthma and asthma samples [189]. While these studies support genetic or epigenetic contributions to enhanced RV-induced remodeling, further investigation is necessary to confirm if RV exaggerates the phenotypes of remodeling-associated alleles, as well as if RV-induced epigenetic programs enhance airway remodeling.

The study of RV-evoked airway remodeling has been limited by model systems available to investigators. Many studies utilize human primary cell culture of epithelium, smooth muscle, and fibroblasts that recapitulate the responses of the individual cell types but does not capture the integrated response of the entire tissue in and around the airway. Medium transfer models have shown success in demonstrating the effect of epithelial RV infection on sub-epithelial cell types. Human airway tissue models such as hPCLS may provide the closest comparison to natural RV infection, though restricted availability and relatively short duration of viability limit their use for longer term studies. Murine models may enable the long-term development of RV-induced airway remodeling, although only certain RV-B serotypes have been consistently utilized in murine infection studies. These serotypes are largely not the same serotypes that induce severe symptoms in non-diseased individuals and have not been shown to be major contributors to severe exacerbations of those with underlying airways disease. Recently, however, two studies have reported RV-C15 – a prevalent strain in humans that may cause severe symptoms – infection of C57BL/6J mice induced the upregulation of remodeling-associated genes *MUC5AC* and *MUC5B*, elevated IL-25, IL-33, and TSLP levels, and increased numbers of lung ILC2s [94, 190]. Given the robust Th2-like response in these animals, the RV-C15 murine infection model demonstrated in these studies may provide a compliant and accessible system for future in vivo investigation or RV-induced airway remodeling.

Targeting RV infection to block airway remodeling-associated signaling has been challenging. Recent attempts to develop highly effective anti-RV therapies have had limited success due to suboptimal efficacy in clinical trials. However, individuals with asthma stand to benefit from RV-targeted therapies given the high risk of asthma exacerbation during RV infection. Meanwhile, ICS treatment continues to be the most efficient means of reducing RV-associated airway inflammation, except in cases of viral-induced steroid resistance. Continued study of airway remodeling evoked during RV infection will provide mechanistic targets and long-term approaches to reduce and reverse irreversible airway obstruction, decreasing rates of viral-associated exacerbations and the overall burden of RV on asthma pathogenesis.

## Data Availability

Not applicable.

## List of abbreviations

α-SMA: α-smooth muscle actin
AEC: airway epithelial cell
AHR: airway hyperresponsiveness
ALI: air liquid interface
ASM: airway smooth muscle
β_2_AR: β_2_ adrenergic receptor
CCL5: C-C motif chemokine ligand 5
CDHR3: cadherin receptor related family member 3
CXCL8: CXC motif chemokine ligand 8
CXCR1: CXC motif chemokine receptor 1
CXCR3: CXC motif chemokine receptor 3
dsRNA: double stranded RNA
ECM: extracellular matrix
EMT: epithelial-to-mesenchymal transition
FGF2: fibroblast growth factor 2
FMT: fibroblast-to-myofibroblast transition
HMVEC-L: lung-derived human microvascular endothelial cell
hPCLS: human precision cut lung slices
ICAM-1: intercellular adhesion molecule 1
ICS: inhaled corticosteroid
IL-4: interleukin 4
IL-13: interleukin 13
IL-25: interleukin 25
IL-33: interleukin 33
ILC2: type 2 innate lymphoid cell
IP-10: interferon γ-induced protein 10 kDa
LDLR: low-density lipoprotein receptor
MMP-9: matrix metalloproteinase 9
MMP-10: matrix metalloproteinase 10
Poly I: C:polyinosinic:polycytidylic acid
PVM: pneumonia virus of mice
RV: rhinovirus
TGFβ: transforming growth factor-β
Th2: T helper cell type 2
TLR3: toll-like receptor 3
TIMP-2: tissue inhibitor of metalloproteinases 2
TNFα: tumor necrosis factorα
TSLP: thymic stromal lymphopoietin
VEGF: vascular endothelial growth factor
